# Do reconstruction filters really effect the volume and ejection fraction calculation with 99^m^Tc-sestamibigated myocardial SPECT?

**DOI:** 10.4103/0972-3919.78251

**Published:** 2010

**Authors:** Ora Manish, Subhash Chand Kheruka, Sukanta Barai, Sanjay Gambhir

**Affiliations:** Department of Nuclear Medicine, SGPGIMS, Lucknow, India

**Keywords:** Butterworth, ejection fraction, Metz, Myocardial perfusion imaging, reconstruction filter

## Abstract

**Background:**

ECG -gated myocardial perfusion imaging is a sensitive test for diagnosis of ischemia as well as scarred myocardium. It provides additional information on global and regional myocardial contractile function. A number of methods are available to calculate left ventricular volumes and ejection fractions, which depends on various technical and patients specific factor.

**Objective:**

This study was carried out to measure effect of reconstruction filter in calculations of left ventricularend diastolic volume (EDV) and end systolic volume (ESV) and left ventricular ejection fraction (LVEF) from 99mTc-sestamibi myocardial perfusion imaging.

**Materials and Methods:**

99mTc-sestamibi-gated SPECT myocardial perfusion imaging was performed in 90 patients. Studies were reconstructed with Butterworth and Metz filters.

**Results:**

Mean ejection fraction with Butterworth and Metz filter are 64.3 and 64.2, respectively. Mean EDV is for Butterworth and Metz filters are 77.3 and 78.5 ml, respectively. While ESV was 36.49 and 36.63 ml, stroke volume calculated was 41.54 and 42 ml for Butterworth and Metz filters, respectively. Pearsons’s correlation coefficients between results calculated with Metz and Butterworth filters were 0.994 for ESV, 0.996 for EDV, 0.966 for LVEF and 0.925 for SV. Student ‘t’ test was applied on the data and no significant difference was noted between parameter estimated by Butterworth or Metz filter.

**Conclusion:**

This study shows that difference of filter application has no significant effect in computing left ventricular function parameters.

## INTRODUCTION

Myocardial perfusion imaging is a sensitive test for diagnosis of ischemia as well as scarred myocardium. Electrocardiography (ECG)-gated imaging providesadditional information on global and regional myocardial contractilefunction,[1] and allows the calculation of left ventricularend diastolic volume (EDV), end systolic volume (ESV), strokevolume (SV) and ejection fraction (LVEF).[2] This functional informationgives additional prognostic information

A number of methods are available to calculate left-ventricularvolumes and ejection fractions.[3,4] Various volumes and ejection fraction calculated depended on software used,[5] calculation method used, acquisition parameter such as number of frames,[6] zoom factor,[7] filter used for image reconstruction.[8,9] The accuracy of results may also be affected by patient-specificfactors such as cardiac volume, patient size and perfusion defectsize.[10]

It has been emphasized that the effect of changing filter may invalidate the clinical parameter. This study was carried out to measure effect of reconstruction filter in calculations of left-ventricularEDV and ESV and LVEF from ^99m^Tc-sestamibi myocardial perfusion imaging.

## MATERIALS AND METHODS

Patients ^99m^Tc-sestamibi-gated SPECT myocardial perfusion imagingwas performed in 90 patients.

### Gated SPECT acquisition

Myocardial gated SPECT was carried out 1 hourafter intravenous injection of 400 MBq ^99m^Tc-sestamibi. SPECTacquisition was carried out on a dual-head large field of view γ-camera (DXTXL SMV). Sixty-four projections (32per head) were obtained in 64×64 matrices using a step andshoot acquisition over a 180° arc from right anterior obliqueto left posterior oblique position. Acquisition zoom was 1.33, giving a pixel size of 6.7 mm. All studies were acquired with 16 framesper cardiac cycleusing an R-wave trigger and a 40% acceptance window.

### Gated SPECT data processing

Studies were processed on a Xeleris version 1.330. Images werepre-filtered, and then reconstructed by filtered back-projectionwith a ramp filter. Two filters recommended by the manufacturerfor reconstruction of gated SPECT studies, Butterworth order10, cut-off frequency 0.394 cycles/pixel and Metz order 3.25, full-width half-maximum 2.35 mm, were compared. The former is a lowpass filter, while the latter is an edge-enhancement filter. Both these types of filter have been shown to be effectivein reconstructing SPECT studies. Myocardial EDV (ml) and ESV (ml), SV (ml) and LVEF (%) weredetermined using a commercial semi-automatic gated SPECT processingsoftware, Emory toolbox. Processing was performedby a single operator for each study using thetwo filters concurrently

### Calculations

Statistical analysis was performed with the SPSS program version13 for Windows. The Spearman rankcorrelation coefficient was used to test for correlations. Theindividual differences for each patient between filtering withMetz and Butterworth filters were calculated, and statisticaldifferences were tested for using a paired *t*-test.

## RESULT

**Figure 1:**
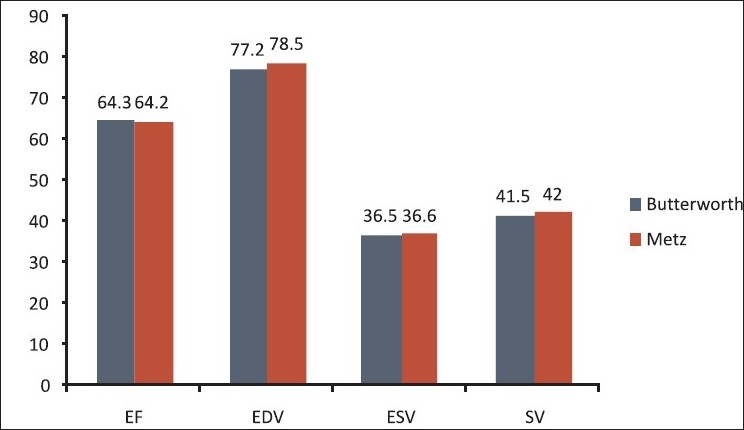
Bar diagram representing different parameter measured from Butterworth and Metz filter

**Figure 2:**
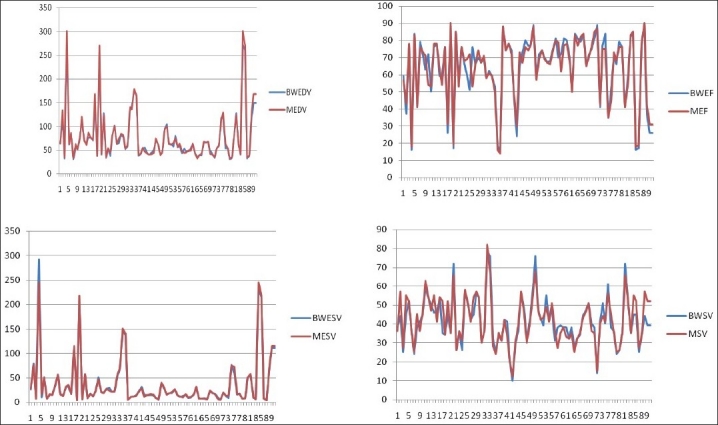
Linear bar diagram to show correlation of EF, EDV, ESV and SV measured by Butterworth and Metz filter

**Figure 3:**
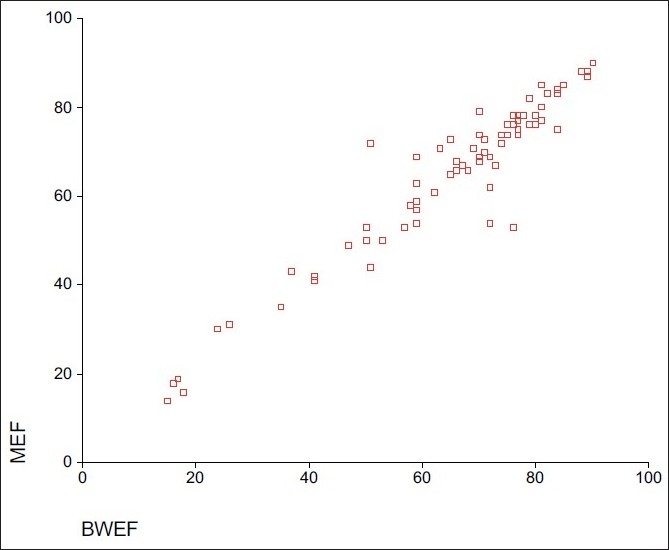
Scatter diagram showing correlation between ejection fraction measured from both filters

The ESV and EDV, SV and LVEF are shown in Table 1. Mean ejection fraction with Butterworth and Metz filter are 64.3 and 64.2. Mean EDV is for Butterworth and Metz filters are 77.3 and 78.5 ml. While ESV was 36.49 and 36.63 ml, SV calculated was 41.54 and 42 ml for Butterworth and Metz filters, respectively. Pearsons’s correlation coefficients betweenresults calculated with Metz and Butterworth filters were 0.994for ESV, 0.996 for EDV, 0.966 for LVEF and 0.925 for SV [Figures 1–3].

**Table 1:** Different cardiac volume measured by Butterworth and Metz filter

	Minimum	Maximum	Mean	Std. deviation
BWEF	15	90	64.32	19.774
BWEDV	31	277	77.23	52.564
BWESV	4	292	36.49	52.388
BWSV	10	78	41.54	12.866
MEF	14	90	64.20	19.049
MEDV	32	300	78.50	56.636
MESV	4	245	36.63	51.183
MSV	12	82	42.04	12.806

BW: Butterworth filter, M: Metz filter, EF: ejection fraction, SV: stroke volume, EDV: end diastolic volume, ESV: end systolic volume

Student ‘*t*’ test was applied on the data and no significant difference was noted between parameter estimated by Butterworth or Metz filter [Table 2].

**Table 2:** Result of Student ‘*t*’ test

	Paired differences					Sig. (2-tailed)
	Mean	Std. deviation	Std. error mean	95% Confidence interval of the difference		
			
				Lower	Upper	
BWEF - MEF	0.122	5.123	0.540	-0.951	1.195	0.821
BWEDV - MEDV	-1.267	6.342	0.668	-2.595	0.062	0.061
BWESV – MESV	-o.144	5.662	0.597	-1.330	1.041	0.809
BWSV – MSV	-0.500	4.961	0.523	-1.539	0.539	0.342

BW: Butterworth filter, M: Metz filter, EF: ejection fraction, SV: stroke volume, EDV: end diastolic volume, ESV: end systolic volume

## DISCUSSION

During processing of ECG gated Myocardiac perfusion imaging quantification starts with thedetection of the LV endocardial and epicardial boundaries. Mostalgorithms first estimate the location of the midmyocardium, which corresponds to the maximal myocardial count. From themidmyocardial points, endocardial and epicardial boundariescan be extracted either by using a fixed number of SDs of gaussianfitting to the myocardial count profile[11,12] or using a predefinedcount threshold based on the phantom data.[13] Once the definitionsof the endocardial and epicardial edges are achieved, LV volumeis calculated by multiplying the number of pixels within theLV cavity with the size of a pixel. LV volume can be generatedfor each of the frames in the cardiac cycle. The largest volumeand the smallest volume represent the EDVand the ESV, respectively. LVEF is derivedfrom the volumes using the formula (EDV – ESV)/EDV ×100.

As endocardial edge is found fromthe maximum slope of the profile between the cardiac centerand the wall center,[14] which would be expected to varywith filter, and to give a thicker wall measurement with a smootherfilter.[15] A thicker wall will lead to a smaller cardiac volume, and so volumes were expected to be smaller with the smootherButterworth filter is comparison to sharper Metz filter. But we were not able to demonstrate this difference is our study. We expected that the difference in wall thickness wouldbe comparable in both end-diastole and end-systole, so the SV wouldnot be affected by the filter that was found true later in study. As both EDV and ESV are not affected by application of different filter so EF is also same.

The result of this study is in contrast with that of observed by Vakhtangandze *et al*,[16] and Wright A[8] *et al*, which have shown that smoother reconstruction filters lead to lower volumes and higher ejection fractions. Although excellent correlation was noted between the filters but the difference was significant statistically. Number of patients was 30 and 40 in these studies. Mean difference of ejection fraction was -3.5 ± 0.9 (Metz and Butterworth) and 2.55 ± 3.10% (Butterworth and Hann filter). This difference may be statistically significant but unlikely to be clinically significant.

LVEF is a has been established as an indicator of prognosisafter myocardial infarction[17] and heart failure.[18] ESV is the most sensitive parameter in determiningimprovement in left ventricular function after revascularization.[19] Left ventricularvolumes can be measured by radionuclide ventriculography.[20] White *et al*, also focusing on patients with recent myocardial infarction demonstrated that besides LVEF, left ventricular volumes are important in the prediction of survival. Progressive increments of 25 ml in ESV augmented the relative risk of cardiac death in an exponential fashion: as compared to patients with a normal ESV (30-55 ml), patients with an ESV of 75 ml and 125 ml had a 2.5-fold and a 5-fold higher relative risk of cardiac death, respectively.[21]

Gated cardiac scan has one additional advantage of increasing specificity of myocardiac perfusion imaging. As perfusion-scan fixed defects may result from soft tissue attenuation, decreasing test specificity for coronary disease and myocardialinfarction (MI). Gated ^99m^Tc-sestamibi SPECT may help differentiateMI from artifact since fixed defects with decreased function(wall motion and thickening) probably represent MI, whereasattenuation artifacts either have normal function or at leastdo not demonstrate markedly reduced function.[22]

## CONCLUSION

There were no statistically significant mean differences in EDV, ESV, LVEF and SV measured using Butterworth and Metz filters. The differences found in our study are dissimilar to those from previous studies comparing reconstruction with different filters.[16,17] The correlations between two filters were good for all functional parameter.

These results show that difference of filter application has no major effect on left ventricular function parameter.
